# Vaccine Effectiveness Among 5- to 17-year-old Individuals with Prior SARS-CoV-2 Infection: An EHR-Based Target Trial Emulation Study from the RECOVER Project

**DOI:** 10.21203/rs.3.rs-6945998/v1

**Published:** 2025-07-03

**Authors:** Yong Chen, Yuqing Lei, Jiajie Chen, Qiong Wu, Ting Zhou, Bingyu Zhang, Michael Becich, Yuriy Bisyuk, Saul Blecker, Elizabeth Chrischilles, Dimitri Christakis, Lindsay Cowell, Mollie Cummins, Soledad Fernandez, Daniel Fort, Sandy Gonzalez, Sharon Herring, Benjamin Horne, Carol Horowitz, Mei Liu, Susan Kim, Parsa Mirhaji, Abu Mosa, Jennifer Muszynski, Catharine Paules, Alice Sato, Hayden Schwenk, Soumitra Sengupta, Srinivasan Suresh, Bradley Taylor, David Williams, Yongqun He, Jeffrey Morris, Ravi Jhaveri, Christopher Forrest

**Affiliations:** Department of Biostatistics, Epidemiology and Informatics, University of Pennsylvania; University of Pennsylvania; University of Pennsylvania; University of Pittsburgh; University of Pennsylvania; University of Pennsylvania; University of Pittsburgh School of Medicine; University Medical Center New Orleans; Department of Population Health, New York University Grossman School of Medicine; Department of Epidemiology, College of Public Health, The University of Iowa; Center for Child Health, Behavior and Development, Seattle Children’s Research Institute; UT Southwestern Medical Center; University of Utah; The Ohio State University; Ochsner Health; Nicklaus Children’s Hospital, Miami, FL; Program for Maternal Health Equity, Center for Urban Bioethics, Department of Population Health and Urban Bioethics, Center for Obesity Research and Education, College of Public Health, Lewis Katz S; Intermountain Medical Center Heart Institute; Icahn School of Medicine at Mount Sinai; University of Florida; University of California, San Francisco; Albert Einstein College of Medicine; University of Alabama at Birmingham; Nationwide Children’s Hospital; Division of Infectious Diseases, Department of Medicine, Penn State Health Milton S. Hershey Medical Center, Hershey, PA; 982162 Nebraska Medical Center, Omaha, NE 68198; Stanford School of Medicine; Columbia University; University of Pittsburgh School of Medicine; Medical College of Wisconsin; University of Michigan Medical School; University of Michigan; University of Pennsylvania; Ann & Robert H. Lurie Children’s Hospital of Chicago; Children’s Hospital of Philadelphia

## Abstract

The effectiveness of COVID-19 vaccination in children and adolescents with prior SARS-CoV-2 infection remains unclear, particularly for Omicron subvariants. We evaluated vaccine effectiveness against reinfection with Omicron BA.1/2, BA.4/5, XBB, and later subvariants among 5- to 17-year-olds using data from the RECOVER initiative, a national electronic health record database covering 37 U.S. pediatric institutions. We emulated target trials by age group and variant period, comparing previously infected participants between January 2022 and August 2023. During the BA.1/2 period, vaccination reduced the risk of reinfection, with effectiveness rates of 62% in children and 65% in adolescents. During the BA.4/5 period, protection effectiveness in children was 57%, whereas no statistically significant protection was observed in adolescents. During the XBB or later period, no significant protection was observed in either group. In summary, COVID-19 vaccination provided protection against reinfection during early and mid-Omicron periods in previously infected pediatric populations, but effectiveness declined for later variants.

## INTRODUCTION

The rapid development and distribution of COVID-19 vaccines against SARS-CoV-2 in response to the global pandemic represent a remarkable scientific and public health achievement. During the early stages of the pandemic, extensive research established the effectiveness of these vaccines in preventing severe disease, hospitalization, and death in individuals without prior SARS-CoV-2 exposure.^1–10^ As the pandemic progressed, numerous variants of SARS-CoV-2 emerged, often causing less severe infections, at least partially because of the widespread population immunity from both vaccination and previous infections. The Centers for Disease Control and Prevention (CDC) has recommended COVID-19 vaccination for individuals with prior SARS-CoV-2 infections^11^, underscoring the potential benefits of hybrid immunity—a combination of infection-acquired and vaccine-induced immunity.

Emerging evidence on hybrid immunity indicates that such individuals often exhibit superior and more durable immune responses compared to those with either immunity type alone^12–19^. However, pediatric studies on hybrid immunity are limited, especially those stratified by SARS-CoV-2 variants. Preliminary findings suggest that hybrid immunity may confer enhanced protection against immune-evasive variants such as Delta, Omicron BA.1, and BA.2, but these findings are predominantly derived from adult populations^20,21^. Notably, a study from Qatar reported that hybrid immunity provided the strongest protection against symptomatic Alpha, Beta, and Delta infections, assuming independent contributions of infection- and vaccine-induced immunity across age groups^22^. However, subsequent analysis found no significant differences in protection among prior infection, vaccination, and hybrid immunity against symptomatic Omicron BA.1 and BA.2 infections^23^. Further insights come from a population-based cohort study^24^ that included participants aged 12 and more, which demonstrated that three or more antigenic exposures — achieved through vaccination or infection — significantly reduced the risk of Omicron-associated hospitalization and death across all age groups. Despite the Omicron variant’s rapid global dissemination since November 2021, research quantifying the effects of hybrid immunity against diverse Omicron subvariants, i.e., B.A. 1/2, B.A. 4/5, XBB, and later, is lacking. Addressing this gap is critical to tailoring vaccination strategies for younger populations as the SARS-CoV-2 landscape continues to evolve.

Despite the global dominance of the Omicron variant since November 2021, research quantifying the effects of hybrid immunity against specific Omicron subvariants—such as BA.1/2, BA.4/5, and XBB—is limited. Addressing this gap is crucial for refining vaccination strategies tailored to younger populations as the SARS-CoV-2 landscape continues to evolve.

This study aims to investigate the understudied population of children and adolescents with prior SARS-CoV-2 infection—a significant group given the delayed vaccine rollout for these age groups, during which many had already acquired natural immunity. Using electronic health record (EHR) data from the Research COVID to Enhance Recovery (RECOVER) Project, we evaluate COVID-19 vaccine effectiveness in previously infected children and adolescents. This study represents the largest to date in the United States on vaccine effectiveness in this population, leveraging data from 37 U.S. children’s hospitals and health institutions. Employing a target trial emulation design, we assess vaccine effectiveness against reinfection, encompassing both asymptomatic and symptomatic cases, and stratify our analysis by different Omicron subvariant periods. To strengthen the robustness of our findings, negative control experiments were performed to adjust for potential unmeasured confounding factors.

The results of this study will provide critical insights for informing vaccination strategies and public health policies targeting pediatric populations with prior infection-induced immunity, ultimately contributing to more effective prevention of future infections in these groups.

## RESULTS

### Statistical Method Overview

This study employed target trial emulation techniques^25–27^ to investigate the effectiveness of the COVID-19 vaccine in preventing SARS-CoV-2 reinfections among previously infected children and adolescents in the United States. To comprehensively assess vaccine effectiveness, we designed six distinct studies stratified by age group (children aged 5–11 years and adolescents aged 12–17 years) and by the prevalence of specific SARS-CoV-2 Omicron subvariants during the study period. This stratification allows for a more precise evaluation of how vaccine protection varies across different viral landscapes and developmental stages. As detailed in Table 1, the six target trials were organized across three major Omicron variant periods.

### Cohort Identification

A total of 48,249 children and 39,324 adolescents from the Omicron BA.1/2 cohort, 123,417 children and 105,909 adolescents from the Omicron BA.4/5 cohort, and 151,961 children and 131,020 adolescents from the Omicron XBB or later cohort within the RECOVER Database were identified to study the effectiveness of vaccination against reinfection during the subsequent variant periods. Baseline characteristics are detailed in Table 2.

Across all cohorts, the majority of both children and adolescents were Non-Hispanic White, with 53.0% of children and 51.4% of adolescents in the B.A.1/2 cohort, 52.1% of children and 50.7% of adolescents in the B.A.4/5 cohort, and 51.2% of children and 50.3% of adolescents in the XBB or later cohort. Hispanic participants were the second-largest group, comprising 20.4% of children and 23.7% of adolescents in the B.A.1/2 cohort, 22.5% of children and 23.8% of adolescents in the B.A.4/5 cohort, and 21.8% of children and 23.5% of adolescents in the XBB or later cohort. The prevalence of obesity was higher among adolescents compared to children, ranging from 43.0% to 46.5% in adolescents and 40.0% to 43.8% in children across cohorts. Most participants (over 83%) had received no prior COVID-19 vaccine doses before the study period. During the study period, 16.6% of children and 9.6% of adolescents in the B.A.1/2 cohort, 9.3% of children and 6.6% of adolescents in the B.A.4/5 cohort, and 7.2% of children and 5.1% of adolescents in the XBB or later cohort received at least one dose of the COVID-19 vaccine. The remaining majority of participants remained unvaccinated throughout the respective study periods. Documented SARS-CoV-2 reinfections during the study period were rare. In the BA.1/2 cohort, reinfections occurred in 2.0% of children and 2.2% of adolescents; in the BA.4/5 cohort, 0.9% of children and 0.9% of adolescents experienced reinfection; and in the XBB or later cohort, 0.6% of children and 0.9% of adolescents had documented reinfections.

### Vaccine Effectiveness

**Figure 1:**
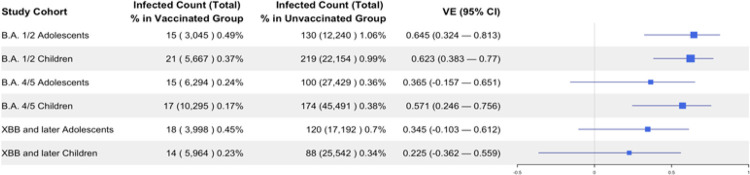
Vaccine Effectiveness Against SARS-CoV-2 Reinfection Across Omicron Subvariants (BA.1/2, BA.4/5, XBB and Later) in Vaccinated and Unvaccinated Children and Adolescents.

The COVID-19 vaccination demonstrated effectiveness in preventing SARS-CoV-2 reinfection among children and adolescents during the Omicron B.A.1/2 and B.A.4/5 periods, but reduced effectiveness was observed during the XBB and later period. Figure 1 summarizes the estimated vaccine effectiveness in sequential trials emulations framework for 6 target trials. For B.A.1/2 study cohort, 8,712 (5,667 children and 3,045 adolescents) were eligible for the vaccinated group for the vaccine effectiveness analysis against B.A. 1/2, and 34,394 (22,154 children and 12,240 adolescents) were matched to unvaccinated controls. For the B.A.4/5 study cohort, 16,589 (10,295 children and 6,294 adolescents) were eligible for the vaccinated group for the vaccine effectiveness analysis against B.A.4/5, and 72,920 (45,491 children and 27,429 adolescents) were matched to unvaccinated controls. For the XBB or later study cohort, 9,962 (5,964 children and 3,998 adolescents) were eligible for the vaccinated group for the vaccine effectiveness analysis against XBB or later, and 42,734 (25,542 children and 17,192 adolescents) were matched to unvaccinated controls. The analysis results showed those who received at least one dose of the COVID-19 vaccine during the study period demonstrated statistically significant protection against BA.1/2 variant infection, with an effectiveness of 62.3% (95% CI: 38.3% to 77.0%) in children aged 5 to 11 years and 64.5% (95% CI: 32.4% to 81.3%) in adolescents aged 12 to 18 years. For the BA.4/5 variant, the vaccine conferred protection of 57.1% (95% CI: 24.6% to 75.6%) in children, while in adolescents, the protection was not statistically significant (36.5%, 95% CI: −15.7% to 65.1%). Additionally, the COVID-19 vaccine did not confer statistically significant protection against the XBB variant in either children (22.5%, 95% CI: −36.2% to 55.9%) or adolescents (34.5%, 95% CI: −10.3% to 61.2%).

### Sensitivity Analysis

The sensitivity analysis was firstly stratified based on the last infected variants of the participants, which yielded that the COVID-19 vaccine provided significant protection against the Omicron BA.1/2 variant in both children and adolescents previously infected with the Delta variant. However, vaccine effectiveness against subsequent Omicron sub-variants, including BA.4/5 and XBB, showed non-significant results, particularly in adolescents. These inconsistencies in vaccine effectiveness are largely because of the low numbers of observed outcomes for these specific variants, which likely impacted the analysis. The detailed results are presented in **Section S3.A** of the Supplementary Appendix.

In the sensitivity analysis assessing vaccine effectiveness in preventing symptomatic COVID-19 illness among children and adolescents with prior SARS-CoV-2 infections, severity outcomes were categorized as asymptomatic, mild, moderate, or severe, following Forrest et al (2022)^28^. The incidence rates are summarized in Supplementary **Table S3**. The analysis showed that the COVID-19 vaccine had positive effectiveness in preventing symptomatic illness during the Omicron BA.1/2 and BA.4/5 periods, as presented in Supplementary **Figure S38**.

Moreover, the sensitivity analysis restricted to Pfizer recipients yielded results consistent with the primary analysis, confirming similar patterns of vaccine effectiveness across all study cohorts (Supplementary **Figure S39**).

Finally, negative control experiments (**Section S3. D**) indicated the presence of a slight positive systematic error, as evidenced by a minor shift in point estimates to the right (Supplementary **Table S7**). This rightward shift suggests a potential deflation of the primary results, meaning the vaccine effectiveness might be slightly underestimated.

## DISCUSSION

This target trial emulation study represents the largest investigation into hybrid immunity in a pediatric cohort, examining the effectiveness of the COVID-19 vaccine in children and adolescents with a history of SARS-CoV-2 infection. Significant protection against the Omicron B.A.1/2 and B.A.4/5 variants was demonstrated, highlighting the importance of vaccination even in those previously infected. Our findings indicate that vaccine effectiveness varied across different Omicron sub-variants, with reduced efficacy, particularly against the newer XBB and later variant. The results underscore the challenges of maintaining vaccine efficacy amidst evolving viral landscapes.

Despite the benefits of vaccination, the persistently low vaccination rates in the pediatric population with prior infection has remained a significant public health concern. This is particularly troubling given the observed differences in vaccine effectiveness between age groups. Children consistently showed higher protection rates than adolescents, which may be attributed to age-related variations in immune responses. Younger children typically exhibit more robust immunological responses compared to older adolescents.^29^ Additionally, the timing of vaccine availability plays a critical role; Pfizer’s vaccine was initially approved for individuals aged 16 and older in December 2020, and eligibility expanded to include children aged 12–15 approximately six months later, in May 2021.^30,31^ As a result, older adolescents may have experienced a longer time interval since their initial vaccinations, potentially contributing to reduced vaccine effectiveness. These results reinforce the need for tailored vaccination strategies that account for the baseline immunity conferred by prior infections.

Hybrid immunity can provide enhanced protection against SARS-CoV-2, including various Omicron subvariants such as B.A.1/2, B.A.4/5, and XBB. The synergistic effect of hybrid immunity is attributed to the broader and more robust immune responses it elicits, including increased antibody diversity and durability, along with heightened T-cell responses. Studies suggest that hybrid immunity leads to a more comprehensive recognition of viral epitopes, enabling effective neutralization even against subvariants with significant mutations in the spike protein.^32,33^ Additionally, evidence indicates that hybrid immunity reduces the risk of severe outcomes and may also protect against long COVID.^34^ However, hybrid immunity can also present challenges. Individuals with prior infections might experience immune system overactivation upon vaccination, potentially leading to heightened inflammatory responses or rare adverse events.^35^ Furthermore, the immunological landscape created by hybrid immunity is complex and variable, influenced by factors such as the timing of infection relative to vaccination and the variant involved in the prior infection.^36^ Understanding these dynamics is crucial for tailoring vaccination strategies, particularly in pediatric populations where immune responses may differ significantly from adults.

Our findings regarding the estimated vaccine effectiveness among prior infected participants were consistent with those reported in other studies.^23,24^ On the other hand, our study’s design did not capture unreported home infections of mild severity. Such cases, if undetected, can lead to an overestimation of vaccine effectiveness. The focus on medically detectable infections only further contributes to this limitation, emphasizing the need for a more comprehensive surveillance and reporting system to capture the full spectrum of COVID-19 infection outcomes. The implied upside of this observation is that infections managed at home and not in any medical setting are likely to be minor, so the estimates of vaccine effectiveness may be inflated due to the lack of mild infection data.

## LIMITATIONS

The study has several limitations. The reliance on electronically recorded health data may omit less severe infection events not requiring medical attention, potentially skewing the perceived effectiveness of the vaccine. Additionally, the focus on medically detectable infections might not fully reflect the actual burden of disease, particularly for mild and asymptomatic infections. Future research should aim to evaluate the long-term effectiveness of COVID-19 vaccines in pediatric populations, particularly in the context of emerging variants. Studies that assess the impact of vaccination on mild and asymptomatic infections are also needed to provide a more complete picture of vaccine-induced protection. Such research will help refine vaccination strategies and inform public health policies designed to control the pandemic among younger populations.

## METHODS

### Data Sources

This study is part of the National Institutes of Health (NIH) funded RECOVER Initiative (https://recovercovid.org/), which aims to learn about the long-term effects of COVID-19. This study included 37 US children’s hospitals and health institutions. The EHR data was standardized to the OMOP Common Data Model (CDM)^37^ and extracted from the RECOVER Database Version S11. More details are available in the Supplementary Materials **Section S1**.

### Study Design and Population

For each target trial, eligibility criteria included children aged 5 to 11 years and adolescents aged 12 to 17 years at the start of the study period. Eligible participants had a documented SARS-CoV-2 infection during a specific variant-dominant period (Delta, BA.1/2, or BA.4/5) before the study start date. Individuals younger than 5 or older than 18 years, or those infected during the Delta-Omicron overlap (December 1–31, 2021), were excluded. First infections were confirmed by a positive polymerase chain reaction (PCR) test, serology, antigen test, clinical diagnosis, a prescription for nirmatrelvir/ritonavir, or documentation of post-acute sequelae of SARS-CoV-2 (PASC).

The intervention of interest was receiving at least one dose of a COVID-19 vaccine during the study period, compared to no vaccination during the study period. Target trial emulation was conducted every 7 days within each study period, creating sequential 7-day trials. Participants could initially belong to the comparator group before receiving vaccination. For each 7-day trial, participants were required to remain uninfected and unvaccinated during the study period up until the trial start date. Vaccinated participants without infection during the trial were assigned to the intervention group, with the index date as their vaccination date. Participants who remained unvaccinated or were infected before vaccination during the trial were placed in the comparator group, with the trial start date as their index date. Participants with both infection-acquired and vaccine-induced immunity during the study period were categorized as having hybrid immunity.

### Outcomes

The primary outcome was documented SARS-CoV-2 reinfection during each study period. Reinfection was identified by a subsequent positive PCR or antigen test, clinical diagnosis, or prescription for nirmatrelvir/ritonavir, occurring at least 60 days after the previous infection. Reinfections within 60 days were considered ongoing infections. Participants were followed from their index date until the earliest of the following: documented SARS-CoV-2 reinfection, matched participant’s reinfection, matched participant’s vaccination, or the study period’s end.

### Statistical Analysis

**Figure 2:**
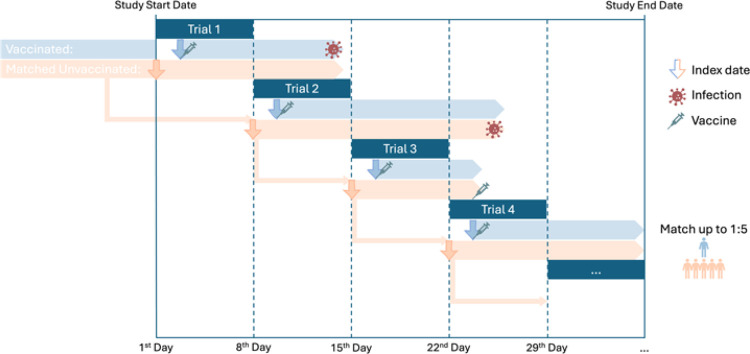
Study design for the emulation of a target trial evaluating the effectiveness of COVID-19 vaccination versus no vaccination in preventing SARS-CoV-2 reinfection among previously infected children and adolescents. Participants were enrolled in sequential 7-day trials, with vaccinated individuals compared to matched unvaccinated controls. The index date was defined as the vaccination date for the intervention group and the trial start date for the comparator group. Up to 1:5 matching with replacement was used.

To balance baseline characteristics, we applied exact matching and propensity score matching to the intervention and comparator groups. We first performed exact matching of each eligible participant who is vaccinated within the 7-days trial period to all eligible participants who were unvaccinated during the trial period using factors including gender, race/ethnicity (Hispanic, Non-Hispanic White, Non-Hispanic Black, Asian American/Pacific Islander, Other), indicators from the data-contributing sites, number of COVID-19 vaccine doses before cohort entry, and time since the last vaccination. Prior infection characteristics, including the variant and time since infection, were also matched. Subsequently, within the exact matching strata, propensity scores were estimated using logistic regression models, adjusting for age, obesity status, and chronic conditions defined by the Pediatric Medical Complexity Algorithm (PMCA)^38^. Conditions included cardiovascular, respiratory, neurological, and other system-specific diseases. Healthcare utilization, medication records, days since the last infection, and prior infection severity were also incorporated. Up to 1:5 (at least 1:1) matching with replacement was used within each nested sequential trial. An unvaccinated comparator could serve as a match for vaccinated persons in more than one sequential trial as long as they remained unvaccinated. Detailed variable definitions are provided in **Table S1** of the Supplementary Appendix. Figure 2 provides a visual demonstration of the target trial emulation design used in this study. It illustrates the sequential 7-day trial framework, where vaccinated individuals were compared with matched unvaccinated controls.

We used Cox proportional hazards models to estimate vaccine effectiveness, defined as 100*(1 - hazard ratio), with 95% confidence intervals (CIs). This analysis examined the relationship between post-infection vaccination and two outcomes: SARS-CoV-2 reinfection and symptomatic reinfection. We emulated 25 target trials for the B.A.1/2 period, 24 for B.A.4/5, and 39 for XBB/later variants, pooling participants across each study period for analysis. Detailed statistical methods are described in **Section S2** of the Supplementary Appendix.

### Sensitivity Analysis

To assess the robustness of our findings, we conducted a series of sensitivity analyses. First, we stratified the analysis by the last infected variant, as detailed in Supplementary Materials **Section S3 part A**. Second, we evaluated vaccine effectiveness against varying severities of COVID-19 illness (mild, moderate, severe), which is reported in **Section S3 Part B**. Additionally, as shown in Supplementary **Figure S19**, the majority of vaccinated participants in the study cohorts received the Pfizer COVID-19 vaccine. Across all cohorts, Pfizer accounted for more than 90% of vaccinations, with only a small proportion receiving Moderna or unspecified vaccines. This dominant use of the Pfizer vaccine justified conducting a sensitivity analysis restricted to Pfizer recipients, which presented in **Section S3 Part C**. Finally, we implemented negative control outcome experiments using a list of 40 negative control outcomes, determined by board-certified pediatricians, to calibrate residual study bias from unmeasured confounders, assuming the null hypothesis of no effect. The empirical null distribution and calibrated risks are presented in **Section S3 Part D**.

All analyses were performed using R software, version 4.1.0 (R Foundation).

## Supplementary Files

This is a list of supplementary files associated with this preprint. Click to download.

- supphybirdimmunity01272025.docx

## Figures and Tables

**Table 1.** Target Trial Design Stratified by Age Group and Prevalent SARS-CoV-2 Omicron Subvariants During the Study Period.

#	Study Cohort	Age Group	Study Period	Prevalent Variants	Variants of Previous Infections
**1**	Omicron B.A. 1/2 Children Cohort	Children (age 5 to 11)	2022/01/01 ~ 2022/06/20	Omicron B.A. 1/2	Delta
**2**	Omicron B.A. 1/2 Adolescents Cohort	Adolescents (age 12 to 17)	2022/01/01 ~ 2022/06/20	Omicron B.A. 1/2	Delta
**3**	Omicron BA. 4/5 Children Cohort	Children (age 5 to 11)	2022/06/21 ~ 2022/11/30	Omicron B.A. 4/5	Delta, Omicron B.A. 1/2
**4**	Omicron BA. 4/5 Adolescents Cohort	Adolescents (age 12 to 17)	2022/06/21 ~ 2022/11/30	Omicron B.A. 4/5	Delta, Omicron B.A. 1/2
**5**	Omicron XBB or later Children Cohort	Children (age 5 to 11)	2022/12/01 ~ 2023/08/30	BQ1, XBB, and later subvariant	Delta, Omicron B.A. 1/2, Omicron B.A. 4/5
**6**	Omicron XBB or later Adolescents Cohort	Adolescents (age 12 to 17)	2022/12/01 ~ 2023/08/30	BQ1, XBB, and later subvariant	Delta, Omicron B.A. 1/2, Omicron B.A. 4/5

**Table 2.** Baseline characteristics of patients before matching.

**Study Period**	**B.A.1/2**		**B.A.4/5**		**XBB or later**	
Age Constraint	Children	Adolescents	Children	Adolescents	Children	Adolescents
**Total**	**48,249**	**39,324**	**123,417**	**105,909**	**151,961**	**131,020**
**Race**						
Asian American/Pacific Islander	1,447	637	5,688	3,727	7,634	5,006
Non-Hispanic Black	8,762	8,157	21,195	18,768	26,026	22,921
Non-Hispanic White	22,639	18,872	52,937	48,451	63,090	58,587
Hispanic	9,847	7,969	27,818	23,216	35,303	29,640
Other	5,554	3,689	15,779	11,747	19,908	14,866
**Sex**						
Male	24,856	19,669	64,343	51,538	79,678	64,242
Female	23,391	19,648	59,068	54,351	72,272	66,755
Unknown	2	7	6	20	11	23
**Obese**						
Obese	20,043	17,452	53,831	47,503	68,151	60,975
Non-Obese	13,018	11,067	40,118	31,618	50,242	39,442
Unknown	15,188	10,805	29,468	26,788	33,928	30,603
**Prior Vaccine Doses**						
0	40,194	32,186	90,863	69,638	110,791	83,924
1	2,998	1,647	4,667	4,008	6,169	5,200
2+	5,057	5,491	27,887	32,263	35,001	41,896
**Vaccination During Study Period**						
**Vaccinated**	6,852	3,779	11,461	7,023	6,760	4,668
Unvaccinated	41,397	35,545	111,956	98,886	145,201	126,352
**Time Since Last Vaccine Prior Study Start Date**						
>= 4month	11	3,079	24,196	30,268	30,678	39,724
<4 Month	8,044	4,059	8,358	6,003	10,492	7,372
Unvaccinated	40,194	32,186	90,863	69,638	110,791	83,924
**Severity of Last Covid Infection**						
Asymptomatic	29,843	24,025	74,835	65,133	88,925	79,036
Mild	16,802	13,524	43,423	36,228	55,513	46,078
Moderate	1,179	1,305	3,954	3,273	5,709	4,258
Severe	425	470	1,205	1,275	1,814	1,648
**Documented Infection During Study Period**						
Infected	986	868	1,094	990	980	1,167
Uninfected	47,263	38,456	122,323	104,919	150,981	129,853

## Data Availability

The data is not publicly available due to privacy concerns. The individual de-identified participant data will not be shared. The data that support the findings of this study may be available through request and DUA process to the RECOVER initiative.

## Code availability

The analysis code is available for interested readers by emailing the corresponding author, Dr. Yong Chen, through ychen123@pennmedicine.upenn.edu.

## References

[1] Wu Q. Real-World Effectiveness of BNT162b2 Against Infection and Severe Diseases in Children and Adolescents. Ann Intern Med 177, 165–176 (2024). 38190711 10.7326/M23-1754 PMC11956830

[2] Wu Q. Real-world effectiveness and causal mediation study of BNT162b2 on long COVID risks in children and adolescents. EClinicalMedicine 79, 102962 (2025). 39720603 10.1016/j.eclinm.2024.102962 PMC11667630

[3] Lopez Bernal J. Effectiveness of Covid-19 Vaccines against the B.1.617.2 (Delta) Variant. New England Journal of Medicine 385, 585–594 (2021). 34289274 10.1056/NEJMoa2108891 PMC8314739

[4] Feldstein L. R. Effectiveness of Bivalent mRNA COVID-19 Vaccines in Preventing SARS-CoV-2 Infection in Children and Adolescents Aged 5 to 17 Years. JAMA 331, 408 (2024). 38319331 10.1001/jama.2023.27022 PMC10848053

[5] Andrews N. Covid-19 Vaccine Effectiveness against the Omicron (B.1.1.529) Variant. New England Journal of Medicine 386, 1532–1546 (2022). 35249272 10.1056/NEJMoa2119451 PMC8908811

[6] Hungerford D. & Cunliffe N. A. Real world effectiveness of covid-19 vaccines. BMJ n2034 (2021) doi:10.1136/bmj.n2034.

[7] Pilishvili T. Effectiveness of mRNA Covid-19 Vaccine among U.S. Health Care Personnel. New England Journal of Medicine 385, (2021). 10.1056/NEJMoa2106599 PMC8482809 34551224

[8] Tartof S. Y. Estimated Effectiveness of the BNT162b2 XBB Vaccine Against COVID-19. JAMA Intern Med 184, 932 (2024). 38913355 10.1001/jamainternmed.2024.1640 PMC11197441

[9] Hansen C. H. Short-term effectiveness of the XBB.1.5 updated COVID-19 vaccine against hospitalisation in Denmark: a national cohort study. Lancet Infect Dis 24, e73–e74 (2024). 38190834 10.1016/S1473-3099(23)00746-6

[10] Brannock M. D. Long COVID risk and pre-COVID vaccination in an EHR-based cohort study from the RECOVER program. Nat Commun 14, 2914 (2023). 37217471 10.1038/s41467-023-38388-7 PMC10201472

[11] https://www.cdc.gov/covid/vaccines/faq.html.

[12] Mongin D. Effect of SARS-CoV-2 prior infection and mRNA vaccination on contagiousness and susceptibility to infection. Nat Commun 14, 5452 (2023). 37673865 10.1038/s41467-023-41109-9 PMC10482859

[13] Matuchansky C. Protection against SARS-CoV-2 after Vaccination and Previous Infection. N Engl J Med 386, 2534 (2022). 35704417 10.1056/NEJMc2205618

[14] Painter M. M. Prior vaccination promotes early activation of memory T cells and enhances immune responses during SARS-CoV-2 breakthrough infection. Nat Immunol 24, 1711–1724 (2023). 37735592 10.1038/s41590-023-01613-y

[15] Sokal A. Maturation and persistence of the anti-SARS-CoV-2 memory B cell response. Cell 184, 1201–1213.e14 (2021). 33571429 10.1016/j.cell.2021.01.050 PMC7994111

[16] Gazit S. The Incidence of SARS-CoV-2 Reinfection in Persons With Naturally Acquired Immunity With and Without Subsequent Receipt of a Single Dose of BNT162b2 Vaccine. Ann Intern Med 175, 674–681 (2022). 35157493 10.7326/M21-4130 PMC8855786

[17] Bobrovitz N. Protective effectiveness of previous SARS-CoV-2 infection and hybrid immunity against the omicron variant and severe disease: a systematic review and meta-regression. Lancet Infect Dis 23, 556–567 (2023). 36681084 10.1016/S1473-3099(22)00801-5 PMC10014083

[18] Yung C. F. BNT162b2 vaccine protection against omicron and effect of previous infection variant and vaccination sequence among children and adolescents in Singapore: a population-based cohort study. Lancet Child Adolesc Health 7, 463–470 (2023). 37201540 10.1016/S2352-4642(23)00101-3 PMC10185330

[19] Gazit S. Hybrid immunity against reinfection with SARS-CoV-2 following a previous SARS-CoV-2 infection and single dose of the BNT162b2 vaccine in children and adolescents: a target trial emulation. Lancet Microbe 4, e495–e505 (2023). 37062294 10.1016/S2666-5247(23)00103-9 PMC10101759

[20] Singanayagam A. Community transmission and viral load kinetics of the SARS-CoV-2 delta (B.1.617.2) variant in vaccinated and unvaccinated individuals in the UK: a prospective, longitudinal, cohort study. Lancet Infect Dis 22, 183–195 (2022). 34756186 10.1016/S1473-3099(21)00648-4 PMC8554486

[21] Andeweg S. P. Protection of COVID-19 vaccination and previous infection against Omicron BA.1, BA.2 and Delta SARS-CoV-2 infections. Nat Commun 13, 4738 (2022). 35961956 10.1038/s41467-022-31838-8 PMC9373894

[22] Altarawneh H. N. Effects of previous infection, vaccination, and hybrid immunity against symptomatic Alpha, Beta, and Delta SARS-CoV-2 infections: an observational study. EBioMedicine 95, 104734 (2023). 37515986 10.1016/j.ebiom.2023.104734 PMC10404859

[23] Altarawneh H. N. Effects of Previous Infection and Vaccination on Symptomatic Omicron Infections. New England Journal of Medicine 387, 21–34 (2022). 35704396 10.1056/NEJMoa2203965 PMC9258753

[24] Wu S. Protection of prior SARS-CoV-2 infection, COVID-19 boosters, and hybrid immunity against Omicron severe illness: A population-based cohort study of five million residents in Canada. PLoS One 19, e0299304 (2024). 38394091 10.1371/journal.pone.0299304 PMC10889649

[25] Hernández-Díaz S. Emulating a Target Trial of Interventions Initiated During Pregnancy with Healthcare Databases: The Example of COVID-19 Vaccination. Epidemiology 34, 238–246 (2023). 36722806 10.1097/EDE.0000000000001562 PMC9891154

[26] Ioannou G. N. Effectiveness of Nirmatrelvir–Ritonavir Against the Development of Post–COVID-19 Conditions Among U.S. Veterans. Ann Intern Med 176, 1486–1497 (2023). 37903369 10.7326/M23-1394 PMC10620954

[27] Hernán M. A. & Robins J. M. Using Big Data to Emulate a Target Trial When a Randomized Trial Is Not Available: Table 1. Am J Epidemiol 183, 758–764 (2016). 26994063 10.1093/aje/kwv254 PMC4832051

[28] Forrest C. B. Severity of Acute COVID-19 in Children <18 Years Old March 2020 to December 2021. Pediatrics 149, (2022). 10.1542/peds.2021-055765 35322270

[29] Nziza N. Humoral profiles of toddlers and young children following SARS-CoV-2 mRNA vaccination. Nat Commun 15, 905 (2024). 38291080 10.1038/s41467-024-45181-7 PMC10827750

[30] https://www.cdc.gov/acip/evidence-to-recommendations/covid-19-pfizer-biontech-etr-12-15-years.html.

[31] https://www.fda.gov/news-events/press-announcements/fda-approves-first-covid-19-vaccine.

[32] The Lancet Infectious Diseases. Why hybrid immunity is so triggering. Lancet Infect Dis 22, 1649 (2022). 36372089 10.1016/S1473-3099(22)00746-0 PMC9648977

[33] Lasrado N. & Barouch D. H. SARS-CoV-2 Hybrid Immunity: The Best of Both Worlds. J Infect Dis 228, 1311–1313 (2023). 37592872 10.1093/infdis/jiad353

[34] Mikolajczyk R. Likelihood of Post-COVID Condition in people with hybrid immunity; data from the German National Cohort (NAKO). Journal of Infection 89, 106206 (2024). 38897239 10.1016/j.jinf.2024.106206

[35] Voss W. N. Hybrid immunity to SARS-CoV-2 arises from serological recall of IgG antibodies distinctly imprinted by infection or vaccination. Cell Rep Med 5, 101668 (2024). 39094579 10.1016/j.xcrm.2024.101668 PMC11384961

[36] Aguilar-Bretones M. SARS-CoV-2-specific immune responses converge in kidney disease patients and controls with hybrid immunity. NPJ Vaccines 9, 93 (2024). 38806532 10.1038/s41541-024-00886-0 PMC11133345

[37] https://www.ohdsi.org/data-standardization/.

[38] Simon T. D., Cawthon M. L., Popalisky J. & Mangione-Smith R. Development and Validation of the Pediatric Medical Complexity Algorithm (PMCA) Version 2.0. Hosp Pediatr 7, 373–377 (2017). 28634166 10.1542/hpeds.2016-0173 PMC5485351

